# Maternal Obesity in Sheep Increases Fatty Acid Synthesis, Upregulates Nutrient Transporters, and Increases Adiposity in Adult Male Offspring after a Feeding Challenge

**DOI:** 10.1371/journal.pone.0122152

**Published:** 2015-04-15

**Authors:** Nathan M. Long, Daniel C. Rule, Nuermaimaiti Tuersunjiang, Peter W. Nathanielsz, Stephen P. Ford

**Affiliations:** 1 Center for the Study of Fetal Programming, University of Wyoming, Laramie, Wyoming, United States of America; 2 Department of Animal and Veterinary Sciences, Clemson University, Clemson, South Carolina, United States of America; 3 Department of Animal Science, University of Wyoming, Laramie, Wyoming, United States of America; 4 Center for Pregnancy and Newborn Research, Department of Obstetrics and Gynecology, University of Texas, Health Sciences Center, San Antonio, Texas, United States of America; INRA, FRANCE

## Abstract

Maternal obesity in women is increasing worldwide. The objective of this study was to evaluate differences in adipose tissue metabolism and function in adult male offspring from obese and control fed mothers subjected to an *ad libitum* feeding challenge. We developed a model in which obese ewes were fed 150% of feed provided for controls from 60 days before mating to term. All ewes were fed to requirements during lactation. After weaning, F1 male offspring were fed only to maintenance requirements until adulthood (control = 7, obese = 6), when they were fed *ad libitum* for 12 weeks with intake monitored. At the end of the feeding challenge offspring were given an intravenous glucose tolerance test (IVGTT), necropsied, and adipose tissue collected. During the feeding trial F1obese males consumed more (P < 0.01), gained more weight (P < 0.01) and became heavier (P < 0.05) than F1control males. During IVGTT, Obese F1 offspring were hyperglycemic and hypoinsulinemic (P < 0.01) compared to F1 control F1. At necropsy perirenal and omental adipose depots weights were 47% and 58% greater respectively and subcutaneous fat thickness 41% greater in F1obese vs F1control males (P < 0.05). Adipocyte diameters were greater (P ≤ 0.04) in perirenal, omental and subcutaneous adipose depots in F1obese males (11, 8 and 7% increase vs. control, respectively). When adipose tissue was incubated for 2 hrs with C-14 labeled acetate, subcutaneous, perirenal, and omental adipose tissue of F1 obese males exhibited greater incorporation (290, 83, and 90% increase vs. control, respectively P < 0.05) of acetate into lipids. Expression of fatty acid transporting, binding, and syntheses mRNA and protein was increased (P < 0.05) compared to F1 control offspring. Maternal obesity increased appetite and adiposity associated with increased adipocyte diameters and increased fatty acid synthesis in over-nourished adult male offspring.

## Introduction

Obesity is epidemic in developed and developing countries including a significant number of women of child-bearing age, including one third of women in the US [1,2,3,4]. In parallel with increased maternal obesity, the childhood obesity rate in Western societies has increased to 15–20% and is predicted to increase further [5,6]. The prevalence of obesity is greatest among children of obese mothers [7]. There is an independent association between maternal BMI and offspring adiposity and insulin resistance [8,9,10]. There is currently much interest in the impacts of in-utero developmental programming during maternal obesity on predisposing offspring to a variety of health problems increased risk of developing metabolic syndrome [8]. Compelling human and animal studies strongly suggest that obesity and other chronic diseases may be programmed during fetal life [11,12,13,14]. In precocial species (e.g. sheep and humans) in contrast to altricial rodents adipogenesis and lipogenesis occur predominantly prenatally [15].

In our ovine model of pre-pregnancy maternal obesity, weight of fetuses of obese mothers increased 30% by mid-gestation in combination with increased fetal circulating glucose, insulin, and IGF-1 and greater mid-gestation pancreatic weights compared with fetuses from mothers fed recommendations, [14]. In addition, at both mid- and late gestation, placental fatty acid transporter expression was upregulated in placentae from obese ewes compared to control ewes, and was positively associated with increased maternal and fetal circulating lipids [15]. As a result, late gestation fetuses from obese ewes showed increased adiposity, increased adipocyte diameters, lipid content, nutrient transporter expression, and fatty acid synthesizing enzymes compared to control fetuses [16]. Newborn lambs born to obese ewes had similar birth weights but greater adiposity compared with lambs born to control ewes [14].

Obese offspring exhibited a decreased ability of insulin to dispose of glucose into body tissues compared to control offspring before a feeding challenge [17]. By the end of an *ad libitum* feeding trial these differences were markedly increased, with obese offspring exhibited increased insulin resistance and a decrease in first-phase insulin secretion compared to control offspring. Further, obese offspring exhibited increased feed intake, body weight gain and fat mass during the feeding trial compared to control offspring. Because dual X-ray absorptiometry was used to measure body fat mass it was not possible to determine whether the increase in body fat after the *ad libitum* feeding challenge was a result of increased visceral or subcutaneous fat depots.

The present study was conducted to evaluate the impact of maternal obesity in altering the size, structure, mRNA and protein expression and function of omental, perirenal, mesenteric and subcutaneous fat depots in adult offspring subjected to an *ad libitum* feeding challenge.

## Materials and Methods

All animal procedures were approved by the University of Wyoming IACUC. Beginning 60 days before conception and continuing through parturition, Rambouillet:Columbia cross ewes (3–5 years of age with 2–3 previous pregnancies) were fed either a highly palatable diet at 100% of National Research Council (NRC) [18] recommendations (Control, Con) or 150% of NRC’s recommended (Obese, OB). All ewes were weighed weekly and rations adjusted for metabolic body weight (BW^0.75^). Body condition was scored monthly to evaluate changes in fatness. A body condition score of 1 to 9 was assigned by two trained observers [19].

The animals utilized in this study resulted from 13 ewes with 2 singles and 5 twin pregnancies in the control ewes and 3 singles and 3 twin pregnancies in the OB ewes. No two offspring were used from the same ewe. During lactation, ewes were fed to NRC requirements. Prior to two weeks of age, male lambs were tail-docked and castrated as per Federation of Animal Science Societies recommendations [20]. Male offspring were generated over 2 consecutive breeding seasons utilizing a single ram. From weaning until 2 to 3 years of age, depending on year of birth, male offspring were maintained as a group and fed to NRC maintenance requirements. Four 3 year old and three 2 year old male Con males and three 3 year old and three 2 year old OB males were adapted from a hay and grain diet to the experimental ration at maintenance levels over a 2 week acclimation period after which they were placed on a 12 week *ad libitum* feeding trial using the same diet previously described [17]. A body condition score was determined at the beginning and at the end of the feeding trail. During the feeding trial, wethers were housed in a single group with free access to water and the experimental ration available via an automated feeding behavior data acquisition system (GrowSafe Systems Ltd., Airdrie, Alberta, Canada). Blood samples (~9 mL) were collected every 2 weeks throughout the feeding trial into heparinized tubes (143 USP units per 9 ml of whole blood) at 0700 hours, and blood was centrifuged at 2,500 x *g* and plasma was collected and stored at -80° C. Body weights were obtained every 2 weeks and at the end of the feeding trial.

To confirm and more fully characterize this significant impact of ad libitum feeding on insulin and glucose dynamics, a jugular venous catheter (Abbocath, 16ga, Abbott Laboratories, North Chicago, IL) and extension set was placed aseptically at the end of the trial, 24 hours prior to conducting an intravenous glucose tolerance test (IVGTT). The neck and shoulders were covered with netting (Derma Science Inc, Princeton NJ) to prevent catheter damage. Offspring were maintained in adjacent individual pens with free access to water. No feed was provided for ~ 18 h prior to and during the IVGGT as previously described [13]. Jugular blood samples (~ 6 ml) were obtained into chilled heparinized tubes at -15, and 0 min relative to infusion of a 0.25 g/kg intravenous glucose bolus (50% dextrose solution; Vedco, St, Joseph, MO) administered over 5 seconds. Blood samples were collected at 2, 5, 10, 15, 20, 30, 45, 60, 90 and 120 minutes after glucose infusion. All blood samples were immediately placed on ice, then processed and frozen as previously described. After the IVGGT, the *ad libitum* experimental ration was continued for an additional 2 to 3 days until necropsy.

Male offspring were euthanized with an overdose of sodium pentobarbital (Beuthanasia-D Special; Schering-Plough Animal Health, Union, NJ). Organs and tissue were removed and weighed. Omental adipose tissue was collected close to the dorsal rumen and the celiac artery, perirenal adipose tissue from around the left kidney close to the hylus, and mesenteric adipose tissue was collect between the cecum and the colon. Subcutaneous adipose tissue was collected from above the 12^th^ rib approximately 4 cm off the midline. All adipose tissue was collected by the same trained investigator for all animals. All adipose tissue samples were collected within 2 minutes of euthanasia and weights recorded. Adipose tissue samples for molecular analyses and fatty acid composition were snap frozen in liquid nitrogen and stored at—80°C. The remaining visceral adipose depots were dissected by the same trained individual for all animals and weighed and total depot weights calculated. Subcutaneous adipose tissue thickness was measured 4 cm off the midline over the 12^th^ rib on the side opposite the analysis sampling site. The whole heart was weighed and left and right ventricular free wall dissected and weighed. Ventricular thicknesses were recorded at three random sites across the ventricular wall and values averaged.

In vitro acetate incorporation into total lipids was performed as previously described [21]. Triplicate 100 mg samples of fresh minced (< 30 min after necropsy) adipose tissue from the perirenal, omental, mesenteric, and subcutaneous depots were incubated for 120 min in 30.0 mL of Krebs-Ringer bicarbonate buffer containing 10 m*M* glucose, 5.0 m*M* acetate and 0.5 μCi ^14^C-acetate (Perkin Elmer Life Sciences). Incubations were conducted in 150 x 25 mm siliconized screw-cap tubes in an orbital shaker waterbath at 37°C. Reactions were terminated by first rinsing tissue slices with a mixture of Krebs-Ringer bicarbonate buffer and 2% BSA twice, and total lipids were extracted overnight with chloroform:methanol:water (1:2:0.8,vol/vol/vol). Radioactivity in the total lipid fraction was determined by liquid scintillation spectroscopy. Acetate incorporation rates are expressed as nanomoles acetate converted to total lipid per minute. To exclude the presence of non-metabolically active tissues, rates are expressed as grams of lipid extracted from tissue used in the assay. The intraassay CV for this procedure was 10.8%.

Adipose tissue from perirenal, omental, mesenteric and subcutaneous depots were fixed in 4% paraformaldehyde, embedded in paraffin and sectioned at 10 μm using a MICROM HM310 microtome (MICROM Inc., Walldorf, Germany) to obtain six sections, 100μm apart from each other. Sections were deparaffinized and stained using Harris Modified Hematoxylin (Fisher Scientific, Fair Lawn, NJ) and Eosin Y (EMD Chemicals, Gibbstown, NJ). Images were visualized using an Olympus BX50 microscope and captured digitally using a Retiga EXiFast camera. Pictures at 40x magnification were taken using QED Imaging software (Media Cybernetics, Silver Spring, MD). Five randomly chosen fields were selected per section for a total of 30 pictures per depot. Pictures were randomly selected for analysis and two fields per section were analyzed for cell diameter by one trained investigator blinded to treatment as previously published using Image J Software (NIH, Bethesda, MD) [16,22]. At least 1000 adipocytes per adipose depot were measured.

Western blotting analysis was conducted with commercial antibodies as previously published [22]. Briefly, protein was extracted from ~ 200 mg of pulverized perirenal, omental, mesenteric and subcutaneous adipose tissue using ice-cold lysis buffer and a homogenizer (polytron). Homogenates were sonicated and clarified by centrifugation. The supernatant was mixed with 2×SDS sample loading buffer and heated to 95° C for 5 min. Protein extracts were separated on 7.5 to 10% SDS-PAGE gels and transferred to nitrocellulose membranes for immunoblotting. Band density was normalized according to the β-actin content.

Total RNA was extracted from ~ 500 mg of pulverized adipose tissue using Trizol reagent (Invitrogen Corp., Carlsbad, CA) and purified by RNA binding mini column (Omega Bio-tek Inc., Norcross, GA). One μg of RNA was used to synthesize single-stranded DNA using QuantiTect Reverse Transcription System (QIAGEN, Inc.). Primer sequences for CD36, FATP1, FATP 4, LPL have been previously published by [15], GLUT 4, and AP2 by [23] and fatty acid synthase (FASN) and Acetyl-CoA carboxylase (ACC) by [24]. Quantification of gene expression for the eight genes was expressed relative to 18S rRNA [25]. Expression of 18S rRNA averaged 22.5 ± 3.2 vs 22.9 ± 3.6 Ct for all adipose tissue from Con and OB offspring.

Fatty acid (FA) composition of adipose depots was determined by gas liquid chromatography [26]. Briefly, 1 mL of a 1.0-mg/mL internal standard solution of tri13:0 (glyceryl-tritridecanoate, Sigma Chemical Co, St. Louis, Mo) in chloroform was added to individual 16 x 125 mm tube and was dried under nitrogen. Then ~ 100-mg samples pulverized adipose tissue was added to a standardized tube in duplicate. Fatty acid methyl esters were prepared using direct transesterification with 0.2 *N* KOH in Methanol [26]. Fatty acid methyl esters were separated using an Agilent 6890 GLC (Agilent Technologies, Inc, Palo Alto, Calif) equipped with a 100 m × 0.25 mm fused silica capillary column (SP-2560, 0.2 μm film thickness, Supelco, Bellefonte, Pa) and flame ionization detector. Fatty acid methyl ester peaks were identified by comparing retention times with fatty acid methyl esters standards (Nu-Check Prep, Inc, Elysian, MN,). Fatty acid methyl esters were evaluated using ChemStation software (Agilent Technologies, Inc). Total FA and specific FA concentrations were determined according to Murrieta et al [26].

Glucose was measured colorimetrically in triplicate (Liquid Glucose Hexokinase Reagent, Pointe Scientific, Inc., Canton, MI) [13]. Mean intra-assay CV was 1.2% and inter-assay CV was 2.8%. Insulin was measured in duplicate by commercial RIA (Siemens Medical Solutions Diagnostics, Los Angeles, CA) within a single assay with an intra-assay CV of 9.2% [13]. Leptin concentrations at the start and at weeks 6 and 12 of the feeding trial were determined in a single assay using a commercial RIA (Multispecies RIA. Linco Research, St. Charles MO) previously validated with an intraassay CV of 5.2% [13].

All maternal body weight and body condition scores and offspring data were analyzed using the GLM procedure of SAS (SAS Inst. Inc., Cary, NC) with treatment in the final model. Prism (GraphPad Software Inc, La Jolla, CA) was used to calculate area under the curve (AUC) for plasma glucose and insulin response curves during the IVGTT. Baseline glucose and insulin concentrations in all samples before glucose infusion were averaged to give baseline concentrations. Biweekly plasma hormone and metabolite concentrations, weekly body weight measurements, and plasma glucose and insulin during the IVGTT were analyzed as repeated measures using MIXED procedure of SAS (SAS Inst. Inc., Cary, NC) with treatment and time and their interaction in the model. Fasting glucose and insulin concentrations and AUC were analyzed using the GLM procedure of SAS with treatment in the model. Year of birth had no effect on maternal (P > 0.57) or offspring data (P > 0.27) and was therefore removed from the final models after being initially included. Data are presented as least square means ± SEM, and differences considered significant at P ≤ 0.05, with a tendency at P ≤ 0.1.

## Results

Ewes on the OB diet increased body weight 31% from diet initiation to mating (72.1 ± 3.7 and 94.7 ± 3.9 kg, respectively; P < 0.05) and 47% and 54% from diet initiation to day 75 and day 135 of gestation, respectively (P < 0.05). Control ewes, whose body weight was not different to that of OB ewes at diet initiation (70.4 ± 3.1 kg), exhibited only non-significant body weight gain from diet initiation to conception (2.7%), day 75 (7.1%) or day 135 (13.1%) gestation. Similarly, body condition scores of OB ewes increased (P = 0.02) from diet initiation to mating (5.0 ± 0.2 and 7.1 ± 0.3, respectively), and further increased (P < 0.05) to 7.9 ± 0.2 by day 75, and 8.7 ± 0.2 by day 135. Con ewe body condition scores remained relatively constant from diet initiation to day 135 of gestation (5.1 ± 0.5). Gestation length tended to be shorter (P = 0.07) for OB ewes compared to control ewes (145 ± 2 vs 149 ± 2 d). Birth weight of male lambs was similar between treatment (5.9 ± 0.3 vs 6.3 ± 0.4 kg Con and OB respectively).

**Fig 1:**
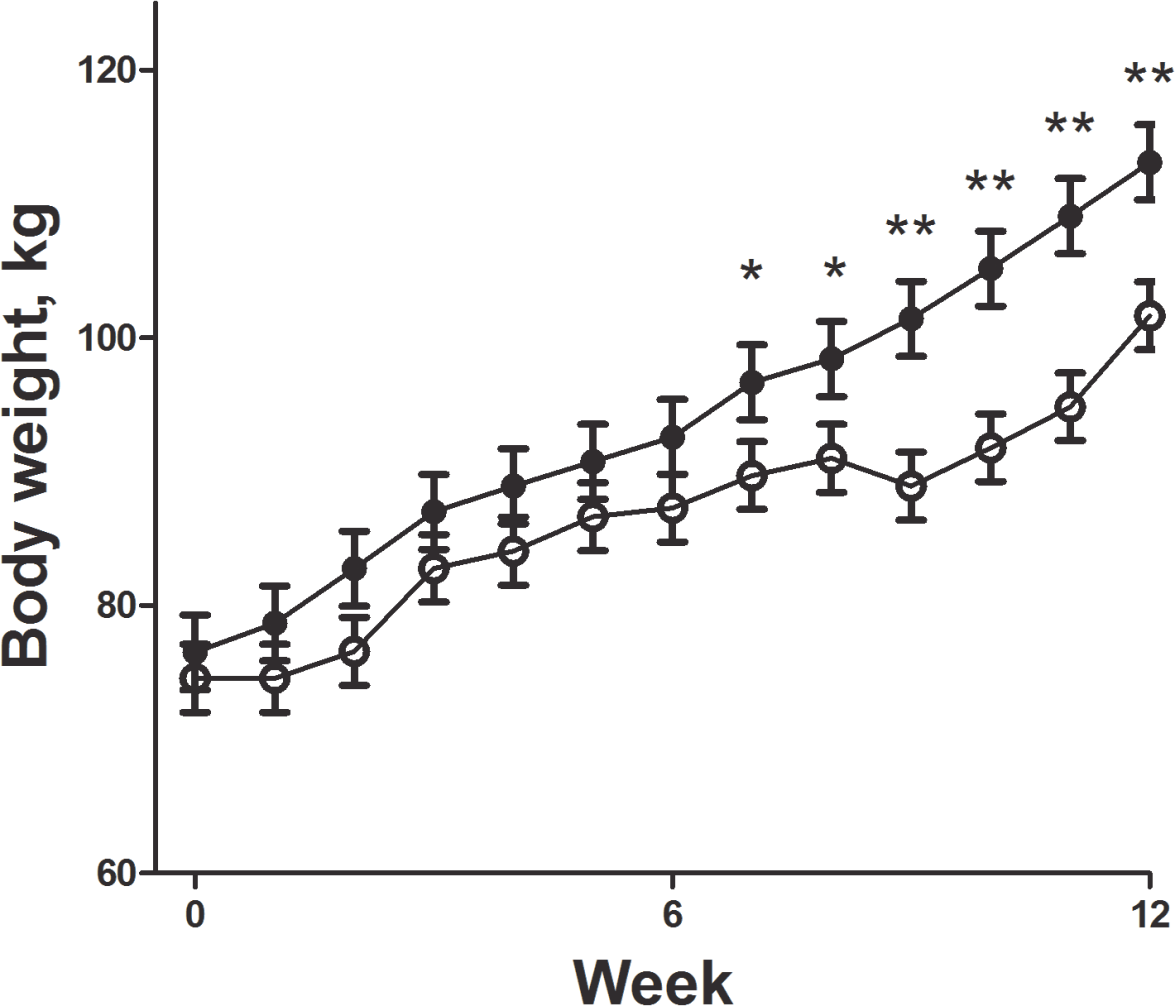
Pattern of weight gain during feeding challenge of male offspring of control ewes (○, n = 7) fed 100% of NRC recommendations and obese ewes (●, n = 6) fed 150% of NRC recommendations during gestation. Values are means ± SEM. Trt P = 0.1669, time P < 0.0001, Trt x time P < 0.0001.* Treatment differences (P < 0.05) and ** Treatment differences (P < 0.01) compared with control.

**Fig 2:**
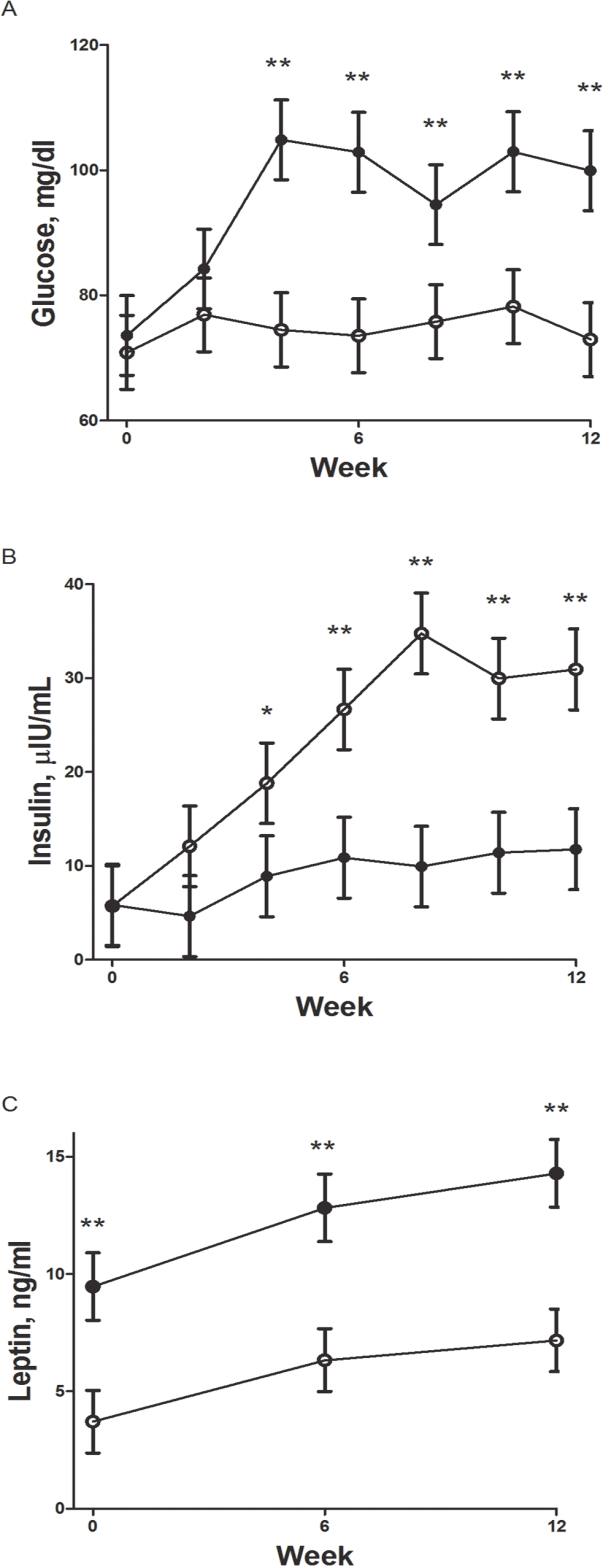
Biweekly plasma concentrations of glucose (a), insulin (b) and leptin (c) during a feeding challenge of male offspring of control ewes (○, n = 7) fed 100% of NRC recommendations and obese ewes (●, n = 6) fed 150% of NRC recommendations. Values are means ± SEM. * Mean differences (P < 0.05) and ** mean differences (P < 0.01) at a specific time point between control and obese offspring.

**Fig 3:**
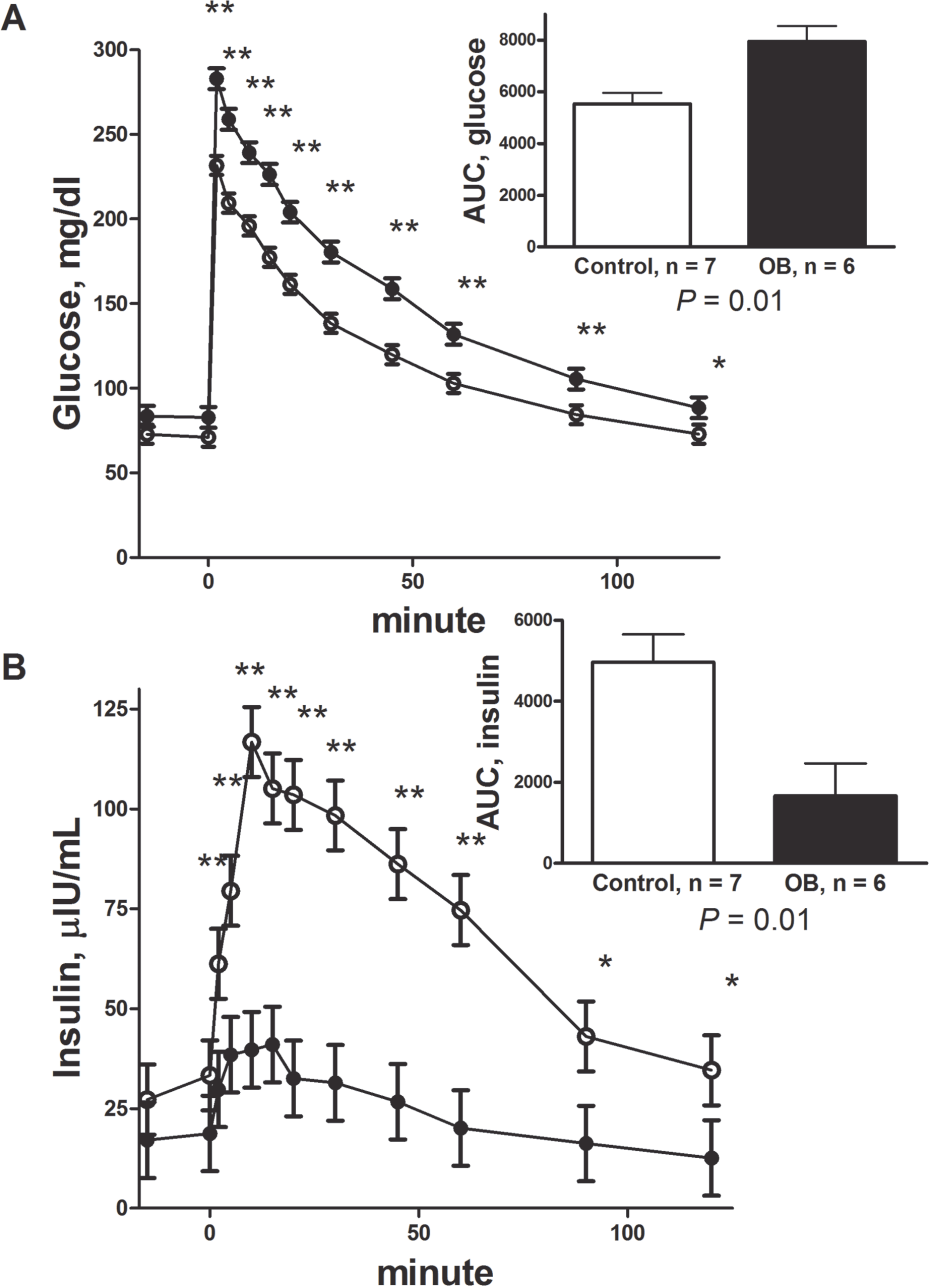
Plasma glucose (a) and insulin (b) responses to a glucose tolerance test in male offspring of control ewes (○, n = 7) fed 100% of NRC recommendations and obese ewes (●, n = 6) fed 150% of NRC recommendations. Area under the curve (AUC) is shown in insert. Values are means ± SEM. a) Trt P < 0.0001, time P < 0.0001, Trt x time P < 0.0001; and b) Trt P = 0.0002, time P < 0.0001, Trt x time P < 0.0001. * Mean differences (P < 0.05) and ** mean differences (P < 0.01) at a specific time point between control and obese offspring.

Body weights of F1Con and F1OB males were similar at the start of the feeding trial (74.57 ± 3.93 vs 73.13 ± 4.19). During the feeding trial, F1OB males ate more feed (257.7 ± 10.2 vs 192.3 ± 9.6 kg respectively P < 0.01) and gained more weight (40.6 ± 2.2 vs 29.5 ± 2.1 kg respectively P < 0.01) than F1Con males (Fig 1). Body condition of F1Con and F1OB males were similar at the beginning of the study (5.2 ± 0.2 vs 5.6 ± 0.2 respectively), while at the end of the feeding trail F1OB males had a greater BCS compared to F1Con males (7.3 ± 0.1 vs 6.9 ± 0.1 respectively; P < 0.01). Plasma glucose concentrations were increased in the biweekly samples from week 4 of the feeding trial until necropsy in F1OB males compared to F1Con males (Fig 2A). Plasma insulin in the biweekly samples was decreased (P < 0.05) from week 4 of the feeding trial until necropsy in F1OB males compared to F1Con males (Fig 2B). Plasma leptin concentrations were increased (P < 0.01) at the beginning and throughout the feeding challenge in F1OB males compared to F1Con males (Fig 2C). The hyperglycemia and hypoinsulinemia of F1OB males compared to F1Con males observed in biweekly samples was also present in fasted samples taken prior to the IVGTT conducted at the end of the feeding challenge (82.59 ± 1.25 vs 71.97 ± 1.10 mg/dl; P < 0.0001 and 17.90 ± 3.10 vs 30.26 ± 2.09 μIU/ml; P < 0.01 glucose and insulin respectively). During the IVGTT, plasma glucose was elevated (Fig 3A) and plasma insulin decreased (Fig 3B) in response to glucose infusion in F1OB males compared to F1Con males.

Body and organs weights and selected measurements obtained at necropsy are given in Table 1. Offspring born to OB mothers weighed more at necropsy than offspring born to Con mothers. The weights of the pancreas, liver, heart, kidney and lung were similar between F1Con and F1OB males. However weight of the right ventricle tended (P = 0.08) to be greater in F1OB males than F1Con males. Similarly, thicknesses of both ventricles were greater (P < 0.05) in F1OB males than F1Con males. Perirenal and omental adipose depot weights were greater (P < 0.04) in F1OB males than F1Con males. The weight of the mesenteric adipose depot tended (P = 0.09) to be greater in F1OB males than F1Con males. The subcutaneous adipose tissue depth over the 12 rib was greater (P = 0.008) in F1OB males than F1Con males. This corresponds to the perirenal and omental adipose depots weights being 47% and 58% increased respectively and subcutaneous fat thickness 41% increased in F1obese compared to the F1control males (P < 0.05).

**Table 1:** Body and organ weights, adipocyte diameters, acetate incorporation of adipose tissue explants of adult male offspring born to control and Obese mothers at necropsy after an *ad libitum* feeding challenge.

	Control	Obese	P Value
Body and organ weights	n = 7	n = 6	
Body weight, kg	104 ± 3	113 ± 3	0.047
Empty Body weight, kg	62.1 ± 2.0	65.1 + 2.3	0.196
Pancreas wt, g	59.38 ± 7.74	56.18 ± 8.36	0.778
Liver wt, g	1926 ± 69	1825 ± 75	0.342
Heart wt, g	457.5 ± 17.0	462.7 ± 18.3	0.841
Right Ventricle wt, g	106.1 ± 4.6	115.1 ± 5.0	0.084
Left Ventricle wt, g	198.9 ± 7.9	205.8 ± 8.0	0.221
Right Ventricle thickness, mm	5.44 ± 0.22	6.14 ± 0.32	0.049
Left Ventricle thickness, mm	13.23 ± 0.34	14.32 ± 0.39	0.032
Average Kidney wt, g	131.9 ± 5.44	135.8 ± 7.42	0.344
Average Lung wt, g	434.9 ± 16.6	420.9 ± 20.6	0.301
Perirenal adipose tissue, g	2503 ± 356	3663 ± 404	0.038
Omental adipose tissue, g	3627 ± 439	5738 ± 628	0.006
Mesenteric adipose tissue, g	1992 ± 221	2489 ± 243	0.092
Subcutaneous adipose tissue thickness, mm	9.06 ± 0.86	12.83 ± 1.11	0.008
Adipocyte diameters			
Perirenal adipose tissue, μm	148.2 ± 8.7	165.1 ± 5.2	0.033
Omental adipose tissue, μm	167.6 ± 3.6	181.0 ± 5.8	0.033
Mesenteric adipose tissue, μm	146.2 ± 5.8	137.6 ± 3.4	0.129
Subcutaneous adipose tissue, μm	116.4 ± 1.9	124.5 ± 4.0	0.041
Acetate incorporation, Mmole/mg tissue/2 hours			
Perirenal adipose tissue	1.55 ± 0.26	2.83 ± 0.04	0.014
Omental adipose tissue,	3.01 ± 0.66	5.72 ± 0.75	0.041
Mesenteric adipose tissue	1.91 ±. 41	2.15 ± 0.34	0.329
Subcutaneous adipose tissue	10.04 ± 1.20	29.07 ± 5.16	0.043

Data are means ± SEM.

Adipocyte diameters were greater (P < 0.05) in omental, perirenal, and subcutaneous adipose depots in F1OB males than F1Con males (Table 1). There was no treatment difference (P = 0.36) in the mesenteric adipose depot adipocyte diameters (Table 1). Concentrations of FA in the adipose tissue depots are given in Table 2. In the subcutaneous adipose tissue, concentrations of 16:0, 18:1 t11, and total FA content were greater (P < 0.05) in F1OB males than F1Con males. In the perirenal and omental adipose tissue concentrations of 16:0, 18:0, and total FA were increased (P < 0.03) in F1OB males compared to F1Con males. In the mesenteric adipose depot concentrations of 16:0 were increased (P = 0.017) in F1OB males compared to F1Con males.

**Table 2:** Fatty Acid composition (mg/g tissue of tissue) from adult male offspring born to control and obese mothers at necropsy after an *ad libitum* feeding period

	Control	Obese	P Value
Perirenal	n = 7	n = 6	
14:0	21.73 ± 1.85	22.11 ± 1.99	0.892
16:0	201.31 ± 6.24	226.52 ± 6.74	0.019
16:1	26.18 ± 2.12	28.73 ± 2.29	0.432
18:0	225.93 ± 7.71	262.67 ± 8.33	0.008
18:1 t11	69.65 ± 8.41	90.91 ± 9.09	0.099
18:1 c9	229.24 ± 9.56	233.80 ± 10.33	0.752
18:2	36.66 ± 2.65	36.38 ± 2.86	0.945
Total FA	906.47 ± 12.95	966.71 ± 13.99	0.009
Omental			
14:0	20.16 ± 1.08	21.27 ± 1.16	0.497
16:0	187.93 ± 6.41	211.74 ± 6.93	0.028
16:1	26.52 ± 1.89	28.81 ± 2.04	0.429
18:0	182.77 ± 8.29	218.45 ± 8.95	0.014
18:1 t11	64.19 ± 11.79	98.89 ± 12.73	0.071
18:1 c9	269.97 ± 7.78	259.52 ± 8.40	0.381
18:2	34.07 ± 2.72	39.35 ± 2.93	0.214
Total FA	884.48 ± 17.19	956.38 ± 18.57	0.016
Mesenteric			
14:0	19.04 ± 0.62	21.50 ± 0.70	0.096
16:0	190.10 ± 4.98	210.67 ± 5.38	0.017
16:1	23.30 ± 1.63	25.08 ± 1.76	0.473
18:0	173.51 ± 11.35	178.41 ± 12.26	0.775
18:1 t11	59.09 ± 8.71	78.46 ± 9.41	0.159
18:1 c9	319.64 ± 9.35	312.34 ± 10.10	0.606
18:2	34.80 ± 2.58	41.86 ± 2.79	0.099
Total FA	912.97 ± 18.68	961.21 ± 20.18	0.097
Subcutaneous			
14:0	9.38 ± 1.05	11.21 ± 1.13	0.261
16:0	102.22 ± 6.25	130.27 ± 6.75	0.011
16:1	42.84 ± 2.70	42.33 ± 2.92	0.901
18:0	62.53 ± 7.56	70.22 ± 8.26	0.508
18:1 t11	29.68 ± 4.18	44.66 ± 4.52	0.033
18:1 c9	202.03 ± 20.76	225.79 ± 22.42	0.453
18:2	23.49 ± 1.37	24.53 ± 1.48	0.616
Total FA	683.71 ± 15.29	734.42 ± 16.51	0.046

Data are means ± SEM.

Table 1 shows incorporation rates of ^14^C labeled acetate into total lipids in tissue explants from the four adipose depots. There was no difference in acetate incorporation, i.e. de-novo fatty acid synthesis rate in the mesenteric depot in tissue explants from F1Con and F1OB males. Omental, subcutaneous (P < 0.05) and perirenal (P < 0.01) adipose fatty acid synthesis were all increased in F1OB males compared to F1Con males (Table 1).

**Fig 4:**
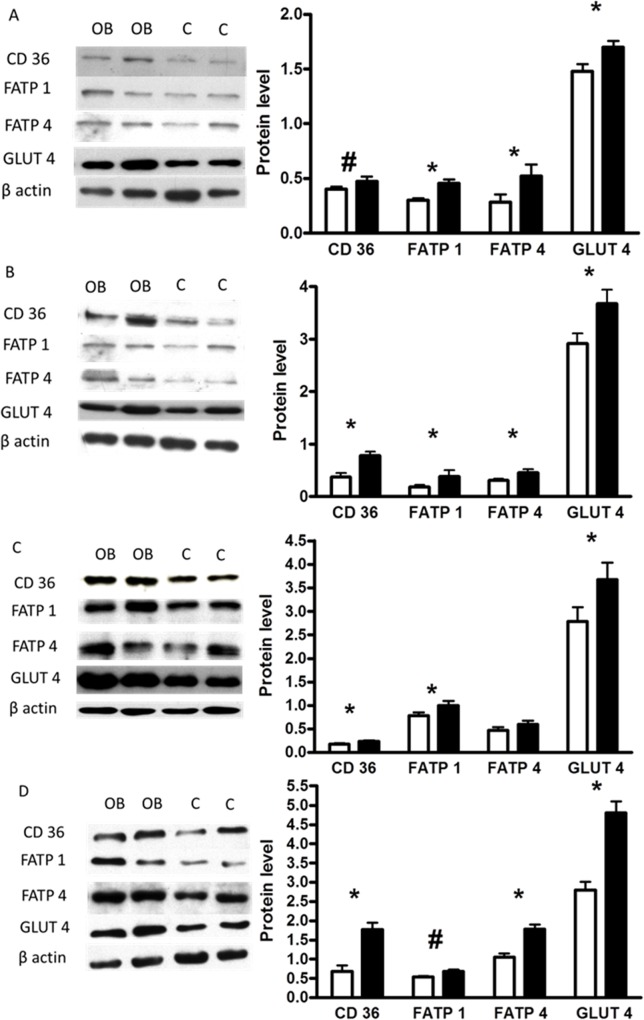
Western blot analysis measurements of fatty acid translocase (CD 36), fatty acid transporter (FATP) 1 and 4, and insulin-sensitive glucose transporter (GLUT4) protein abundance in perirenal (a), omental (b), mesenteric (c), and subcutaneous (d) adipose tissue of male offspring of control ewes (open histogram; n = 7) fed 100% of NRC recommendations and obese ewes (closed histograms; n = 6) fed 150% of NRC recommendations. Values are means ± SEM. # means tend to differ (P < 0.10), * treatment differences (P < 0.05) and ** treatment differences (P < 0.01)

Abundance of mRNA for eight selected genes in the four depots is shown in Table 3. All depots showed a similar offspring response to maternal obesity and overnutrition. Abundance of FASN, ACC, FATP 1 and 4, CD36, AP2, GLUT 4 and LPL mRNA was increased (P < 0.05) in all adipose tissue of F1OB males compared to adipose tissue of F1Con males. Protein abundance of CD36, FATP 1 and 4, and GLUT 4 was increased (P < 0.05) in the omental adipose depot in F1OB males compared to F1Con males (Fig 4A). In the perirenal adipose depot protein abundance of FATP 1 and 4, and GLUT 4 was increased (P < 0.05) in OB males compared to Con males. In the mesenteric adipose depot, protein abundance of CD36, FATP 1, and GLUT 4 increased (P < 0.05) in tissue from F1OB males compared to F1Con males (Fig 4C). In the subcutaneous adipose tissue depot, CD36, FATP 4, and GLUT 4 were all increased (P < 0.05) in subcutaneous adipose tissue of F1OB males compared to F1Con males (Fig 4D).

**Table 3:** Messenger RNA abundance of fatty acid synthase and acetyl-CoA carboxylase, nutrient transporters and associated molecules in adipose tissue depots from adult male offspring born to control and obese mothers at necropsy after an *ad libitum* feeding period

	Control	Obese	P Value
Perirenal	n = 7	n = 6	
Fatty acid synthase (FASN)	4.29 ± 0.92	56.25 ± 12.53	< 0.001
Acetyl-CoA carboxylase (ACC)	9.05 ± 3.51	124.62 ± 7.49	0.003
Fatty acid transporter 1 (FATP1)	6.52 ± 2.19	56.74 ± 17.64	0.004
Fatty acid transporter 4 (FATP4)	23.86 ± 7.08	114.12 ± 10.58	0.012
Fatty acid translocase (CD36)	35.06 ± 12.93	163.76 ± 10.64	0.004
Insulin-sensitive glucose transporter (GLUT4)	22.21 ± 2.38	110.72 ± 5.39	0.004
Lipoprotein lipase (LPL)	18.93 ± 1.83	163.51 ± 14.57	0.022
Fatty acid binding protein 4 (AP2)	11.19 ± 4.23	28.05 ± 3.98	0.016
Omental			
Fatty acid synthase (FASN)	76.81 ± 4.97	184.20 ± 17.94	0.001
Acetyl-CoA carboxylase (ACC)	33.70 ± 9.94	335.22 ± 18.55	0.003
Fatty acid transporter 1 (FATP1)	188.89 ± 11.95	360.63 ± 13.98	0.004
Fatty acid transporter 4 (FATP4)	178.57 ± 15.96	438.13 ± 30.63	0.001
Fatty acid translocase (CD36)	56.17 ± 5.54	122.93 ± 14.97	0.026
Insulin-sensitive glucose transporter (GLUT4)	81.17 ± 15.03	151.31 ± 9.35	0.019
Lipoprotein lipase (LPL)	15.02 ± 7.33	61.56 ± 13.71	0.048
Fatty acid binding protein 4 (AP2)	19.12 ± 3.29	43.17 ± 8.84	0.008
Mesenteric			
Fatty acid synthase (FASN)	10.00 ± 4.61	52.38 ± 13.97	0.017
Acetyl-CoA carboxylase (ACC)	8.98 ± 4.85	47.27 ± 12.63	0.007
Fatty acid transporter 1 (FATP1)	15.40 ± 7.20	332.30 ± 19.31	0.005
Fatty acid transporter 4 (FATP4)	7.43 ± 3.73	29.69 ± 9.43	< 0.001
Fatty acid translocase (CD36)	11.90 ± 4.05	224.83 ± 14.80	< 0.001
Insulin-sensitive glucose transporter (GLUT4)	22.59 ± 8.42	371.40 ± 35.81	0.013
Lipoprotein lipase (LPL)	71.12 ± 5.76	185.20 ± 14.09	0.015
Fatty acid binding protein 4 (AP2)	25.79 ± 2.47	316.37 ± 14.93	0.010
Subcutaneous			
Fatty acid synthase (FASN)	12.58 ± 1.22	66.67 ± 6.61	0.014
Acetyl-CoA carboxylase (ACC)	30.49 ± 3.62	238.58 ± 23.83	0.032
Fatty acid transporter 1 (FATP1)	9.25 ± 3.16	80.39 ± 5.74	0.005
Fatty acid transporter 4 (FATP4)	13.73 ± 1.35	22.58 ± 1.42	0.009
Fatty acid translocase (CD36)	39.23 ± 3.88	474.67 ± 20.04	0.009
Insulin-sensitive glucose transporter (GLUT4)	26.03 ± 2.55	80.45 ± 7.97	0.009
Lipoprotein lipase (LPL)	95.63 ± 9.34	798.3 ± 31.53	0.002
Fatty acid binding protein 4 (AP2)	26.88 ± 2.60	55.72 ± 5.00	0.004

Data are means ± SEM.

## Discussion

The F1OB male offspring had increased perirenal, omental and subcutaneous adipose depots. The effects of maternal obesity on individual adipose depots has not been previously reported to be influenced by maternal obesity beginning before and throughout gestation. In rats maternal obesity did not enhance adipose depots weights at weaning [27] this could be due to the age of the offspring at necropsy in this study. It also appears that these depots effects reported here may have originated in fetal life [16]. We have previously shown that maternal obesity in the ewe results in increased fetal adiposity, increased adipocyte diameters, and decreased fetal muscle mass in late gestation associated with increased mRNA and protein expression of fatty acid and glucose transporters and enzymes that regulate fatty acid synthesis and altered lipid profiles and total lipid in a depot specific manner [16]. Data presented here confirm that the adipose tissue phenotype observed in late gestation fetuses gestated by OB ewes persists until adulthood in the presence of a short period of *ab libitum* intake. In this study we report increased adipocyte diameters, and increased expression of fatty acid transporters and associated proteins, along with glucose transporters. In addition we observed both increased expression of fatty acid synthesis enzymes and fatty acid synthesis rates. Offspring from OB ewes had increased concentrations of 16:0, at least one C18 isomer, and total FAs in subcutaneous, perirenal, and omental adipose depots compared to Con offspring. This altered adipose tissue fatty acid composition in OB offspring indicates increased rates of fatty acid synthesis since the fatty acid biosynthesis pathway terminates with a 16-carbon fatty acid which was increased in all adipose depots. The 16:0 FA can then be elongated to 18-carbon fatty acids and then desaturated into 18:1 *c-*9 or *t*-11. It is therefore significant that we found at least one of these 18 carbon FA increased in adipose depots. It should also be noted that the increased feed intake of the OB offspring is confounded with the maternal effects and could have partially contributed to the greater adipose tissue FA synthetic activity in addition to the regulatory changes. However, this is the normal condition for most ad libitum studies.

To our knowledge, this is the first study in a large precocial animal species demonstrating that offspring from mothers OB at conception and throughout pregnancy develop pancreatic insufficiency in adulthood when subjected to an ad libitum feeding bout. Insulin and glucose homeostasis was significantly altered in F1OB males in response to the *ad libitum* feeding challenge as previously reported [17]. In F1OB males compared to F1Con males, we observed increased plasma basal fasting glucose from weeks 4 to 12 of the feeding challenge as well as during the IVGTT. The increased plasma glucose was accompanied by decreased plasma insulin during weeks 4 to12 of the feeding challenge and minimal insulin release during the IVGTT in F1OB males indicating a diet-induced hypofunctional pancreatic insulin response to glucose. In this model of maternal overnutrition and obesity we have previously reported markedly reduced pancreatic β-cell numbers by late gestation in OB fetuses [28]. Additionally, as offspring became young adults, we found slight increases in insulin resistance in F1OB versus F1Con males with only minor decreases in insulin release to i.v. glucose infusion during IVGTT [17,29]. These data confirm that even prior to the ad libitum feeding bout, there were mild alterations in glucose:insulin dynamics, as we have previously reported [17].

Maternal overfeeding in the rat, mouse, and sheep has been shown to lead to altered appetite in the postnatal offspring [17,30,31,32]. Maternal obesity at conception and throughout gestation in rodents and sheep leads to increased body weight and adiposity in offspring compared with offspring from normal weight mothers [17,31,32,33]. Leptin plays a central role in appetite regulation in rodents and other species [34]. Leptin resistance is one potential mechanism producing obesity and is a key event in the onset of negatively altered energy homeostasis [34,35]. We observed elevated leptin concentrations in OB offspring compared with Con offspring at the beginning and throughout the feeding challenge. Although OB offspring had elevated plasma leptin they showed greater feed intake indicating maternal obesity induced leptin resistance. The mechanism of this leptin resistance is unknown. Leptin resistance has multiple causes which can include altered signaling in leptin target neural circuitry, altered transport of leptin into the brain, and even inflammation in the hypothalamus [2,36,37,38,39,40]. Postnatal lambs from obese ewes fail to exhibit the plasma leptin surge seen in lambs from normal weight ewes, and exhibit an increased postnatal appetite [17,41]. Because adult offspring from these obese mothers have increased appetite, they gained more weight during an *ab libitum* feeding challenge and this increased weight gain appears to be mostly adipose tissue [17]. A modified postnatal leptin surge has also been observed in offspring of obese rodents [42,43,44,45,46,47]. Both increased and deceased neonatal leptin concentrations lead to increased feed intake and bodyweight gain [47,48]. Further, leptin has been reported to have direct effects on glucose metabolism [49] and could be playing a part in the insulin and glucose dysregulation we observed.

In conclusion, these data clearly show that diet-induced maternal obesity during pregnancy in the ewe leads to metabolic alterations that persist into adult life. More specifically, OB offspring have increased appetite, glucose dysregulation and insulin secretion insufficiency, and leptin resistance that appear to increase as these animals get older as evidence by greater responses reported here compared to that of these animals than noted before [17]. The limited numbers of animals and also the two ages of animals although non-significant for any effects on any of the measurements are limitations to the current study. In addition maternal obesity results in persistent programming of adipose tissue with increased adipocyte size, increased nutrient transporter expression, and increased FA synthesis first observed in late gestation. Maternal obesity results in offspring with multiple characteristics of metabolic syndrome and these effects become more marked with advanced age.

## Supporting Information

S1 Table (PDF)

## References

[1] ACOG committee opinion number 315. Obesity in pregnancy. Obstet Gynecol 2005;106: 671–675. 16135613 10.1097/00006250-200509000-00054

[2] Callaway LK, Prins JB, Chang AM, Mclntyre HD. The prevalence and impact of overweight and obesity in an Australian obstetric population. Med J Aust 2006;184: 56–59. 16411868 10.5694/j.1326-5377.2006.tb00115.x

[3] Ogden CL, Carroll MD, Curtin LR, McDowell MA, Tabak CJ, Flegal KM. Prevalence of overweight and obesity in the United States, 1999–2004. JAMA 2006;295: 1549–1555. 16595758 10.1001/jama.295.13.1549

[4] Villamor E, Cnattingius S. Interpregnancy. Weight change and risk of adverse pregnancy outcomes: A population-based study. Lancet 2006;368: 1164–1170. 17011943 10.1016/S0140-6736(06)69473-7

[5] Lobstein T, Frelut ML. Prevalence of overweight among children in Europe. Obes. Rev. 2003;4: 195–200. 14649370 10.1046/j.1467-789x.2003.00116.x

[6] Ogden CL, Flegal KM, Carroll MD, Johnson CL. Prevalence and trends in overweight among us children and adolescents, 1999–2000. JAMA 2002;288: 1728–1732. 12365956 10.1001/jama.288.14.1728

[7] Whitaker RC. Predicting preschooler obesity at birth: the role of maternal obesity in early pregancy. Pediatrics 2004;114: e29–e36. 15231970 10.1542/peds.114.1.e29

[8] Boney CM, Verner A, Tucker R, Vohr BR. Metabolic syndrome in childhood: association with birth weight, maternal obesity and gestation diabetes mellitus. Pediatrics 2005;115: E290–E296. 15741354 10.1542/peds.2004-1808

[9] Dorner G, Plagemann A. Perinatal hyperinsulinism as possible predisposing factor for diabetes mellitus, obesity and enhanced cardiovascular risk in later life. Horm Metab Res 1994;26: 213–221. 8076902 10.1055/s-2007-1001668

[10] Schaefer-Graf UM, Pawliczak J, Passow D, Hartmann R, Rossi R, Buhrer C, et al Birth weight and parental BMI predict overweight in children from mothers with gestational diabetes. Diabetes Care 2005;28: 1745–1750. 15983329 10.2337/diacare.28.7.1745

[11] Armitage JA, Poston L, Taylor PD. Developmental origins of obesity and the metabolic syndrome: the role of maternal obesity. Front Horm Res 2008;36: 73–84. 10.1159/0000115355 18230895

[12] Budge HD, Mostyn J, Evens A, Watkins Y, Sullivan R, Ingleton C, et al Differential effects of fetal number and maternal nutrition in late gestation on prolactin receptor abundance and adipose tissue development in the neonatal lamb. Pediatric Research 53: 2003;302–308. 12538790 10.1203/01.PDR.0000047653.73271.C4

[13] Ford SP, Hess BW, Schwope MM, Nijland MJ, Gilbert JS, Vonnahme KA, et al Maternal undernutrition during early to mid-gestation in the ewe results in altered growth, adiposity,and glucose tolerance in male offspring. J Anim Sci 85: 2007;1285–1294. 17224460 10.2527/jas.2005-624

[14] Ford SP, Zhang L, Zhu M, Miller MM, Smith DT, Hess BW, et al Maternal obesity accelerates fetal pancreatic β-cell but not α-cell development in sheep: prenatal consequences. Am J Physiol Regul Integr Comp Physiol 2009;297: R835–R843. 10.1152/ajpregu.00072.2009 19605766 PMC2739779

[15] Zhu MJ, Ma Y, Long NM, Du M, Ford SP. Maternal obesity markedly increases placental fatty acid transporter expression and fetal blood triglycerides at midgestation in the ewe. Am J Physiol Regul Integr Comp Physiol 2010;229: R1224–R1231. 10.1152/ajpregu.00309.2010 20844260 PMC2980459

[16] Long NM, Rule DC, Zhu MJ, Nathanielsz PW, Ford SP. Maternal obesity upregulates fatty acid and glucose transporters and increases expression of enzymes mediating fatty acid biosynthesis in fetal adipose tissue depots. J Anim Sci 2012,90: 2201–2210. 10.2527/jas.2011-4343 22266999

[17] Long NM, George LA, Uthlaut AB, Smith DT, Nijland MJ, Nathanielsz PW, et al Maternal obesity and increased nutrient intake before and during gestation in the ewe results in altered growth, adiposity, and glucose tolerance in adult offspring. J Anim Sci 2010;88: 3546–3553. 10.2527/jas.2010-3083 20622177

[18] National Research Council. Nutrient Requirements of Sheep National Academy Press, Washington, DC 1985.

[19] Sanson DW, West TR, Tatman WR, Riley ML, Judkins MB, Moss GE. Relationship of body composition of mature ewes with condition score and body weight. J Anim Sci 1993;71: 1112–1116. 8505241 10.2527/1993.7151112x

[20] FASS. Guide for the Care and Use of Agricultural Animals in Agricultural Research and Teaching. Third edition Fed Anim. Sci. Soc., Savoy, IL, 2010.

[21] Rule DC, Beitz DC, Hood RL. A note on the effect of adipocyte size on in vitro lipogenesis from acetate and lactate in subcutaneous adipose tissue of large- and small-frame beef steers at 6 months of age. Anim Prod. 1987;44: 454–456.

[22] Long NM, Tousley CB, Underwood KR, Paisley SI, Means WJ, Hess BW, et al Effects of early to mid gestational undernutrition with or without protein supplementation on offspring growth, carcass characteristics, and adipocyte size in beef cattle. J Anim Sci 2012;90: 197–206. 10.2527/jas.53907 21908644

[23] Long NM, Prado-Cooper MJ, Krehbiel CR, Desilva, U, Wettemann RP. Effects of nutrient restriction of bovine dams during early gestation on postnatal growth, carcass and organ characteristics, and gene expression in adipose tissue and muscle. J Anim Sci 2010;88: 3251–3261. 10.2527/jas.2009-2512 20525929

[24] Ducket SK, Pratt SL, Pavan E. Corn oil or corn grain supplementation to steers grazing endophyte-free tall fescue. II. Effects on subcutanous fatty acid content and lipogenic gene expression. J Anim Sci 2009;87: 1120–1128. 10.2527/jas.2008-1420 19028850

[25] Lomax MA, Sadiq F, Karamanlidis G, Karamitri A, Trayhurn P, Hazlerigg DG. Ontogenic loss of brown adipose tissue sensitivity to β-adrenergic stimulation in the ovine. Endocrinol 2007;148: 461–468. 10.1210/en.2006-0918 17023522

[26] Murrieta CM, Hess BW, Rule DC. Comparison of acidic and alkaline catalysts for preparation of fatty acid methyl esters from ovine muscle with emphasis on conjugated linoleic acid. Meat Sci 2003;65: 523–529. 10.1016/S0309-1740(02)00244-9 22063245

[27] Borengasser SJ, Zhang Y, Kang P, Lindsey P, Ronis MJJ, Bader TM et al Maternal obesity enhances white adipose tissue differentiation and alters genomic-scale DNA methylation in male rat offspring. Endocrinol 2013;154: 4113–4125. 10.1210/en.2012-2255 23959936 PMC3800750

[28] Zhang L, Long NM, Hein SM, Ma Y, Nathanielsz PW, Ford SP. Maternal obesity in ewes results in reduced fetal pancreatic β-cell numbers in late gestation and decreased circulating insulin concentrations at term. Dom Anim Endo 2011;40: 30–39. 10.1016/j.domaniend.2010.08.004 PMC3008620 20933362

[29] Yan X, Huang Y, Zhao JX, Long NM, Uthlaut AB, Zhu MJ et al Maternal obesity-impaired insulin signaling in sheep and induced lipid accumulation and fibrosis in skeletal muscle of offspring. Biol Reprod 2011;85: 172–178. 10.1095/biolreprod.110.089649 21349823 PMC3123384

[30] Muhlhausler BS, Adam CL, Findlay PA, Duffield JA, McMillen IC. Increased maternal nutrition alters development of the appetite-regulating network in the brain. FASEB J 2006;20: 1257–1259. 16684802 10.1096/fj.05-5241fje

[31] Nivoit P, Morens C, Van Assche FA, Jansen E, Poston L, Remacle C, et al Established diet-induced obesity in female rats leads to offspring hyperphagia, adiposity and insulin resistance. Diabetologia 2009;52: 1133–1142. 10.1007/s00125-009-1316-9 19288075

[32] Samuelsson A, Matthews PA, Argenton M, Christie MR, McConnell JM, Jansen EH, et al Diet-induced obesity in female mice leads to offspring hyperphagia, adiposity, hypertension, and insulin resistance. Hypertension 2008;51: 383–392. 18086952 10.1161/HYPERTENSIONAHA.107.101477

[33] Caluwaerts S, Lambib S, van Bree R, Peeters H, Vergote I, Verhaeghe J. Diet-induced obesity in gravid rats engenders early hyperadiposity in the offspring. Metabolism Clin. and Experimental 2007;56: 1431–1438. 17884457 10.1016/j.metabol.2007.06.007

[34] Schwartz MW, Woods SC, Porte D, Seeley RJ, Baskin DG. Central nervous system control of feed intake. Nature 2000;404: 661–671. 10766253 10.1038/35007534

[35] Munzberg H, Myers MG. Molecular and anatomic determinations of central leptin resistance. Nat Neurosci 2005;8: 566–570. 15856064 10.1038/nn1454

[36] El-Haschimi K, Pierroz DD, Hileman SM, Bjørbaek C, Flier JS. Two defects contribute to hypothalamic leptin resistance in mice with diet-induced obesity. J Clin Invest 2000;105: 1827–1832. 10862798 10.1172/JCI9842 PMC378516

[37] Morris DL, Rui L. Recent advances in understanding leptin signaling and leptin resistance. Am J Physiol Endocrinol Metab 2009;297: E1247–E1259. 10.1152/ajpendo.00274.2009 19724019 PMC2793049

[38] Posey KA, Clegg DJ, Printz RL, Byun J, Morton GJ, Vivekanandan-Giri A, et al Hypothalamic proinflammatory lipid accumulation, inflammation, and insulin resistance in rats fed a high-fat diet. Am J Physiol Endocrinol Metab 2009;296: E1003–E1012. 10.1152/ajpendo.90377.2008 19116375 PMC2681305

[39] Schwartz M W, Peskind E, Raskind M, Boyko EJ, Porte D Jr. Cerebrospinal fluid leptin levels: relationship to plasma levels and to adiposity in humans. Nat Med 1996;2: 589–593. 8616722 10.1038/nm0596-589

[40] Zhang X, Zhang G, Zhang H, Karin M, Bai H, Cai D. Hypothalamic IKKbeta/NF-kappa B and ER stress link overnutrition to energy imbalance and obesity. Cell 2008;135: 61–73. 18854155 10.1016/j.cell.2008.07.043 PMC2586330

[41] Long NM, Ford SP, Nathanielsz PW. Maternal obesity eliminates the neonatal lamb plasma leptin peak. J Physiology 2011;589: 1455–1462. 10.1113/jphysiol.2010.201681 21262878 PMC3082103

[42] Delahaye F, Breton C, Risold PY, Enache M, Dutriez-Casteloot I, Laborie C, et al Maternal perinatal undernutrition drastically reduces postnatal leptin surge and affects the development of arcuate nucleus proopiomelanocortin neurons in neonatal male rat pups. Endocrinol 2008;149: 470–475. 10.1210/en.2007-1263 18006626

[43] Elias CF, Lee C, Kelly J, Aschkenasi C, Ahima RS, Couceyro PR, et al Leptin activates hypothalamic CART neurons projecting to the spinal cord. Neuron 1998;21: 1375–1385. 9883730 10.1016/s0896-6273(00)80656-x

[44] Elmquist JK, Ahima RS, Elias CF, Flier JS, Saper CB. Leptin activates distinct projections from the dorsomedial and ventromedial hypothalamicá nuclei. Proceedings of the National Academy of Sciences 1998;95: 741–746. 10.1073/pnas.95.2.741 PMC18491 9435263

[45] Kirk SL, Samuelsson AM, Argenton M, Dhonye H, Kalamatiano T, Poston L. Maternal obesity induced by diet in rats permanently influences central processes regulating food intake in offspring. PLOS ONE 2009;4: e5870 10.1371/journal.pone.0005870 19516909 PMC2690656

[46] Proulx K, Clavel S, Nault G, Richard D, Walker CD. High neonatal leptin exposure enhances brain GR expression and feedback efficacy on the adrenocortical axis of developing rats. Endocrinol 2001;142: 4607–4616. 11606425 10.1210/endo.142.11.8512

[47] Yura S, Itoh H, Sagawa N, Yamamoto H, Masuzaki H, Nakao K, et al Role of premature leptin surge in obesity resulting from intrauterine undernutrition. Cell Metabolism 2005;1: 371–378. 16054086 10.1016/j.cmet.2005.05.005

[48] Toste FP, de Moura EG, Lisboa PC, Fagundes AT, de Oliveira E, Passos MCF. Neonatal leptin treatment programs leptin hypothalamic resistance and intermediary metabolic parameters in adult rat. Br J Nutr 2006;95: 830–837. 16571164 10.1079/bjn20061726

[49] Morton GJ, Schwartz MW. Leptin and central nervous system control of glucose metabolism. Physiol Rev 2011;91: 389–411. 10.1152/physrev.00007.2010 21527729 PMC3379883

