# Supplementary material for: Maternal Obesity in Sheep Increases Fatty Acid Synthesis, Upregulates Nutrient Transporters, and Increases Adiposity in Adult Male Offspring after a Feeding Challenge
Source: PLoS One. 2015 Apr 15;10(4):e0122152. doi: 10.1371/journal.pone.0122152 (PMC4398357; doi:10.1371/journal.pone.0122152)
Supplement: S1 Table — (PDF) [file pone.0122152.s001.pdf]

Supplementary Table 1. Sequences of primers used for RT-PCR

	Forward	Reverse	Reference
Fatty acid synthase (FASN)	GCATCGCTGGCTACTCCTAC	GTGTAGGCCATCACGAAGGT	[24]
Acetyl-CoA carboxylase (ACC)	AGCTGAATTTTCGCAGCAAT	GGTTTTCTCCCCAGGAAAAG	[24]
Fatty acid transporter 1 (FATP1)	ACTGTCTGCCCCTGTACCAC	GGCTGGCTGAAAACTTCTTG	[15]
Fatty acid transporter 4 (FATP4)	GGCACCAACGACAAGAAGAT	GCTCGTCCATCACTAGCACA	[15]
Fatty acid translocase (CD36)	CAAGAAAAATGGGGTGCAAT	CTGGCATTAGAATCCCTCCA	[15]
Insulin-sensitive glucose transporter (GLUT4)	TCACCTTAGTCTCGGTGTTCTTGG	AGATGGCCACAATGGAGACATAGC	[23]
Lipoprotein lipase (LPL)	TGTGCCGCCTTGGGTTCAGC	GCACACGCTCAGAGCCAGCA	[15]
Fatty acid binding protein 4 (AP2)	AAGCTGCACTTCTTTCTCACC	GACCACACCCCCATTCAAAC	[23]
